# Evidence on sulfadoxine-pyrimethamine resistance molecular markers from India: interpret with caution

**DOI:** 10.1186/s12936-024-05027-5

**Published:** 2024-07-24

**Authors:** Nimita Deora, Abhinav Sinha

**Affiliations:** 1 ICMR-National Institute of Malaria Research, New Delhi, India; 2 Academy of Scientific and Innovative Research, Ghaziabad, India

**Keywords:** Malaria, Sulfadoxine-pyrimethamine resistance, Molecular markers, India, Interpretation

## Abstract

**Background:**

Sulfadoxine-pyrimethamine (SP), as a partner to artesunate as ACT is the treatment of choice for uncomplicated *P. falciparum* infections in the majority of India and SP-resistance has a potential to lead to ACT failure. In the lack of robust surveillance of therapeutic efficacy of SP, validate molecular markers of SP-resistance offer a hint of failing SP. However, studies reporting these validated markers often suffer from certain pitfalls that warrant a careful interpretation.

**Main body:**

Critical analyses of the results and their reported interpretations from a recent study and other studies conducted on the WHO-validated molecular markers of SP-resistance in India were analysed and the main problems with studying and reporting of these markers are presented here. It was noted that almost all studies analysed flawed either on the usage, estimation and/or interpretation of the standardized classification of the studies SP mutations. These flaws not only impart spatiotemporal incomparability of the published data but also have the potential of being misunderstood and wrongly translated.

**Conclusion:**

Based on this universal problem in studying, reporting and interpreting the data from the studies on molecular markers of SP-resistance, it is stressed that the future studies should be conducted with utmost caution so that robust evidence may be generated and correctly translated to policy.

## Background

Drug resistance to currently recommended artemisinin-based combination therapy (ACT) for *Plasmodium falciparum* malaria remains the foremost threat to malaria elimination in India [1–3]. India has a dual drug policy for uncomplicated *P. falciparum* malaria: artemether+lumefantrine (AL) in its north-eastern states and artesunate and sulfadoxine-pyrimethamine (AS+SP) in the rest of the country. Although AS+SP treatment failures are reported to be below 10% in India, there are many isolated reports mentioning a high prevalence of mutations in the molecular markers linked to SP-resistance [3–5]. This could be particularly thwarting in the light of recent flags raised for partial artemisinin resistance from eastern India [1, 2]. Therefore, monitoring the efficacy of partner drug (SP) is of pivotal importance, more so when a routine molecular surveillance for drug resistance is not in place in India.

The genotypic-phenotypic association between *P. falciparum* dihydrofolate reductase (*Pfdhfr*) and dihydropteroate synthase (*Pfdhps*) genetic markers and SP-resistance has been validated in malaria-endemic areas [6]. Therefore, the spatiotemporal presence of these markers offers the earliest evidence for SP-resistance and may predict ensuing treatment failures, provided that the data are cautiously analysed. In this context, the most recent paper by Singh et al*.* [7] gains significance by generating such valuable evidence. This paper was critically analysed [7] and a cautionary note was raised that might be useful to global researchers in reporting such data as it may lead to some serious misinterpretations.

## Main text

A lack of usage of the standard World Health Organization (WHO) suggested nomenclature for *Pfdhfr* and *Pfdhps* combination of mutations [8, 9] was noted. The paper compares the self-reported burden of ‘triple’ mutations (*Pfdhfr* C59R+S108N and *Pfdhps* G437A) against the WHO-suggested combination (*Pfdhfr* S108N+N51I+C59R) with other papers [10–15] using the phrase “similar triple mutants in central India”. It is important to note that the data from Chhattisgarh [10] and Madhya Pradesh [11] found no such triple mutations as referred by Singh et al. [7]. On the other hand, the study by Mishra et al*.* [12] was a multi-state study involving 27 sites across India and although they reported 10 triple mutants out of 342 samples (3% as compared to this study’s 74%), it is uncertain how many of them were from central India and hence is not a logical comparative statement. Furthermore, only 6 out of the reported 10 were WHO-suggested triple mutants (*Pfdhfr* S108N+N51I+C59R). Mishra et al*.* [13], data from Madhya Pradesh used a different triple mutation classification (*Pfdhfr* C59R+S108N+I164L) as used in this study (*Pfdhfr* C59R+S108N and *Pfdhps* G437A) and reported a triple mutant prevalence of 1.3% as against 74% reported by Singh et al*.* [7]. Although Pathak et al*.* [14, 15] used the same combination for triple mutant as Singh et al*.* [7] and not the WHO classification, the sample size was low (n = 91) for Pathak et al*.* [14] and (n = 78) for Pathak et al*.* [15]. Further, Pathak et al*.* reported a triple mutant prevalence of 8% in 2014 [14] and 10% in 2020 [15], much lower than that reported in the current study by Singh et al. [7]. Similarly, the quadruple mutants reported by Singh et al. [7] are actually the WHO triple mutants. Therefore, there appears to be an ascertainment bias—conferring high resistance to a falsely classified triple or quadruple mutant and vice versa. As readers often do not pay attention to the ‘type’ of mutants clubbed in triple or quadruple mutants, this leads to a flawed conclusion as has happened in this paper [7]. The authors emphasize that triple mutants (*Pfdhfr* C59R+S108N and *Pfdhps* G437A) are possible resistant forms, but in fact such a combination is the WHO double mutant.

Further, in the paper published by Singh et al*.* [7] the prevalence of mutants also appears to be misestimated. The reported burden of the misclassified triple mutant is 74% whereas the WHO-classified triple mutant was only observed in 3% samples (6/199) thus overestimating both the burden and magnitude of possible resistance. The reported burden of quadruple mutants was 4/235 and it is unclear how the authors reached to a denominator of 235 when only 199 and 168 samples were successfully analysed for *Pfdhfr* and *Pfdhps*, respectively.

Besides, there were numerous instances of fallibility, such as the ambiguous and inconsistent use of the nomenclature of *Pfdhps* mutant at codon 437, inconsistencies in describing the number of samples finally used in the analyses, and the false assertion that the frequency of mutations linked to SP-resistance is “increasing” in the studied area. It is also unclear as to what was the reference time point considered for such a reported increase. The comparison of data from Singh et al*.* [7] and their cited references do not support the claimed increase. In addition, a meta-analysis of published data on SP-resistance markers in India from 2008 onwards did not get sufficient data from Madhya Pradesh to conclude that such mutations are on the rise [5].

## Conclusion

Considering the lack of robust evidence advocating a shift from AS+SP to AL throughout India, such pieces of evidence might mean a lot, if analysed and interpreted correctly. This is even more important if the research is done in rural and tribal areas where the pieces of evidence are already scanty. Therefore, it is suggested that the forthcoming research reports consider these words of caution and try to avoid them as far as possible so that the results are not only robust but standardized for comparisons across time and space.

## Data Availability

No datasets were generated or analysed during the current study.

## Abbreviations

ACT: Artemisinin-based combination therapy
AL: Artemether+lumefantrine
AS+SP: Artesunate and Sulfadoxine-pyrimethamine
*Pfdhfr*: *P. falciparum* Dihydrofolate reductase
*Pfdhps*: *P. falciparum* Dihydropteroate synthase
WHO: World Health Organization
